# GEDI canopy cover assessment using airborne LiDAR across eco-climate regions of the United States

**DOI:** 10.3389/fpls.2026.1951245

**Published:** 2026-09-29

**Authors:** Abid Nazir, Niall P. Hanan, Trevor Roberts

**Affiliations:** 1 Plant and Environmental Sciences, New Mexico State University, Las Cruces, NM, United States; 2 Department of Forest and Wildlife Ecology, University of Wisconsin, Madison, WI, United States

**Keywords:** co-locate, cover, ecosystem and biodiversity, GEDI, LiDAR - remote sensing, relocate

## Abstract

Information about woody plant density and canopy cover is central to our definition of terrestrial ecosystem types and crucial for understanding ecosystem state, dynamics, biogeochemical processes, and biodiversity. The Global Ecosystem Dynamics Investigation (GEDI) LiDAR on the International Space Station (ISS) samples 3-D structural information on woody vegetation structure, including an estimate of canopy cover in each 25 m diameter footprint. This study presents an evaluation of the GEDI fractional canopy cover estimates by comparing GEDI retrievals to high-resolution airborne laser scanning (ALS) data at 20 study sites with ~460,000 GEDI footprints across the United States. Based on the statistical assessment, we observed high correlations (R^2^ = 0.84 without geolocation correction; R^2^ = 0.96 with geolocation correction) with a Root Mean Square Error (RMSE) of 14.1% (without geolocation correction) and 7.2% (with geolocation correction). However, error in GEDI L2B canopy cover in some eco-climate regions is still significant, particularly in drylands (mean relative error (MRE) = 69.8%) and shrublands (MRE = 84.1%). The analysis also determined that the GEDI fractional cover retrievals are most effective when actual canopy cover is low (<20%) or high (>70%), with errors typically inflated in areas where canopy cover is in the 20-70% range. This study provides a baseline evaluation of GEDI canopy cover accuracy across eco-climate regions of CONUS, supporting improved interpretation and future refinement of the GEDI canopy cover product.

## 1 Introduction

Woody plant canopy cover is a biophysical measurement of vegetation structure that is central to how we define ecosystems (e.g., grasslands, woodlands, and forests) while also providing landscape-scale insights into terrestrial processes, ecological and biogeochemical processes and biodiversity (Bonan and Doney, 2018; Croft et al., 2020; Goetz et al., 2015; Lewis et al., 2015; Tang et al., 2019). Canopy cover is also a key variable for forest assessment to provide structural metrics, clumping index, and leaf area index (LAI) (Fang et al., 2019a; Fang, 2021; Verger et al., 2009, Kahiu and Hanan, 2018). Canopy cover is defined as the fraction of the horizontal ground surface covered by tree or shrub canopies (vertical projection) (Rautianen et al., 2015; Specht, 1983). Canopy cover does not typically include understory or herbaceous vegetation growing between or underneath trees (Jennings et al., 1999; Korhonen et al., 2006; Li L. et al., 2023).

Field measurements of canopy cover are frequently utilized in conjunction with other forest inventory data. However, field-based cover measurements are laborious to collect over broad geographic regions (Malhi et al., 2006; Pan et al., 2011) such that satellite-based canopy cover estimates are commonly used at regional to global scales (e.g., Hansen et al., 2013). Canopy cover can be estimated in the field as the summed crown area per unit ground area or calculated by subtracting canopy gap fraction (Pgap) in the vertical projection from 1, where Pgap can be estimated using digital cover/hemispherical photography (DCP/DHP), high resolution airborne or satellite imagery (Hansen et al., 2013; Bera et al., 2023; Chianucci, 2016; Ma et al., 2017; Zhao et al., 2018). Satellite-based canopy cover measurements have been successfully applied for many applications, such as deforestation hotspot detection, and assessment of canopy cover gains and losses (Achard et al., 2014; Estoque et al., 2021; Hansen et al., 2013). However, canopy cover extraction from passive remote sensing is compromised due to the potential for visible reflectance to saturate at higher cover, difficulties in separating dense herbaceous vegetation from tree crowns and the complicated vertical structure of forested landscapes (Fang et al., 2019b; Tang et al., 2019; Verger et al., 2009). For optical remote sensing, clouds, canopy shadows, herbaceous cover, slope attenuation, and signal saturation might also contribute to uncertainty (Armston, 2009; Asner and Warner, 2003; Riaño et al., 2003; Song and Woodcock, 2003).

Direct measurement of 3-dimensional canopy structure through light detection and ranging (LiDAR) is an appealing option to estimate canopy cover that should, in theory, be relatively insensitive to the problems encountered using visible remote sensing. Vertical plant stratification can be detected with LiDAR from plot to the landscape scale (Detto et al., 2015; Lovell et al., 2003; McManamon, 2019). LiDAR remote sensing has been effectively employed in forests to estimate canopy cover (Armston et al., 2013; Goetz and Dubayah, 2011; Hopkinson and Chasmer, 2009; Korhonen et al., 2011; Lefsky et al., 2002; Morsdorf et al., 2006) (Hilker et al., 2010; Korhonen et al., 2011; Rosette et al., 2009; Tang et al., 2016).

ICESat was the first spaceborne LiDAR mission launched in 2003 and a follow-up mission ICESat-2 was launched in 2018 to monitor global ice sheet and glacier mass (Neuenschwander and Magruder, 2019). However, while both ICESat LiDAR systems have been used for vegetation structure retrievals, neither provides extensive information about vegetation structure, particularly cover. The Global Ecosystem Dynamics Investigation instrument (GEDI), deployed on the International Space Station (ISS) in 2018, is the first mission designed specifically to measure vegetation structure (Dubayah et al., 2020). GEDI is a full-waveform laser-ranging system which has recorded over 5 years of data across ±51.6° latitude, as determined by the orbital coverage of the ISS (Li X. et al., 2024). GEDI operates at a wavelength of 1064 nm and collects near-nadir observations in a footprint of 25 m diameter (Dubayah et al., 2020). The ability of GEDI “power” and “coverage” lasers to retrieve information on canopy vertical structure depends on waveform pulse strength, atmosphere attenuation, and solar illumination which impact detection of the land surface through the canopy and beam sensitivity (Dubayah et al., 2020; Hancock et al., 2019). For example, power beams can detect the ground through 98% canopy cover while the lower intensity coverage beams operate up to 95% canopy cover (Dubayah et al., 2020). GEDI provides canopy cover and PAI (plant area index) in the L2B product, and canopy relative height (RH) in the L2A product. These products have facilitated several studies covering forest regrowth and degradation (East et al., 2023; Milenković et al., 2022), aboveground biomass (AGB) mapping (Liang et al., 2023; Silva et al., 2021), and canopy height mapping (Potapov et al., 2021; Sothe et al., 2022).

Uncertainty and precision of GEDI products have been evaluated in multiple ecosystems across the globe (Guillevic et al., 2012; Lei et al., 2022; Li L. et al., 2023). For instance, GEDI-derived forest structure metrics were validated using ALS estimations from both the leaf-off and leaf-on seasons (Mandl et al., 2023). The canopy height product and PAI have also been validated using ALS point cloud data as a reference (Dorado-Roda et al., 2021; Li L. et al., 2023; Liu et al., 2021; Nazir et al., 2026; Brown et al., 2023; Wang et al., 2023a, 2023). However, canopy cover validation studies are more limited (but see, for example, Li X. et al., 2024). Here we argue that the GEDI canopy cover product must be more thoroughly validated, for distinct climatic conditions and for different plant functional types (PFT), to more fully assess its current utility and potential enhancements and applications.

Effective use of canopy cover products requires a thorough understanding of their prediction accuracy in various forest types (Fang et al., 2012; Guillevic et al., 2014; Morisette et al., 2006). Uncertainties in the precise location of the ISS mean that GEDI geolocation errors of ~10 m have been reported (Dubayah et al., 2020; Ni et al., 2021). In the context of approaches to validate GEDI vegetation structure retrievals, several authors have concluded that local-scale (e.g., 10–20 m) misregistration of field or airborne data relative to the GEDI footprint can lead to inaccurate comparisons and misleading validation findings, particularly for heterogeneous canopy cover regions (Li L. et al., 2023; Cui et al., 2021; Storey et al., 2014; Wagner Wolff et al., 2022; Wang et al., 2020). In contrast to spaceborne LiDAR, the geolocation error of ALS data is significantly lower (~1 m) (Padró et al., 2019; Pang et al., 2016). Geolocation errors in GEDI data are normally less than its footprint size (12.5 m radius) (Tang et al., 2023). However, large geolocation differences between aerial and satellite data can cause significant discrepancies, particularly in heterogeneous regions (Ni et al., 2021; Wu et al., 2023; Zhao et al., 2009).

The goal of this study is to use ALS reference data over a sample of sites across diverse biomes of the United States to validate the GEDI canopy cover product. We compare ALS-derived canopy fractional cover (CFC) with GEDI-derived CFC using the GEDI L2B product in 20 sites over the United States (US). CFC accuracy was assessed for different bioclimates and plant functional types (PFT). We also evaluate the impact of local co-registration of GEDI and ALS data for error reduction and assess the most common angle and distance of geolocation offsets in case these might indicate consistent bias (or offsets) in geolocation errors.

## 2 Materials and methods

### 2.1 Study area

We selected 20 sites in the Continental US (CONUS), covering diverse eco-climate systems (**Figure 1**, **Supplementary Table S1**). Several criteria were used to choose sites, including: 1) publicly accessible GEDI and airborne LiDAR datasets; 2) representation of diverse forest types, including Broadleaf Forest (BLF), Needleleaf Forest (NLF), and Shrublands; 3) range of plant height, canopy cover, and terrain slope; 4) areas characterized by natural landscapes (i.e. avoiding major urban and agricultural areas); and 5) sites representing the diverse ecoclimatic zones of CONUS, from arid to humid. The research sites are well dispersed in terms of geolocation, elevation, canopy height, canopy cover, and plant functional type (PFT), enabling a comparative assessment of the strengths and limitations of the GEDI mission in diverse topographical, climatic, and cover conditions. The area of the sites ranges from 300 to 2,300 km², with a total of 12,784 km² for all sites, latitudes from 32°N to 46°N, longitudes from 122°W to 74°W, and elevations from near sea level to 2,344 m above sea-level. Mean annual temperatures across these 20 sites range from 3.6 °C to 19.5 °C, with mean annual precipitation (MAP) between 111 and 1576 mm, and aridity index (AI = MAP/PET) between 0.19 and 7.1. The spatial extent of individual study sites covers hundreds of square kilometers ranged between 300 and 2,400 km². The relatively extensive ALS coverage at each site provided a spatially continuous, high resolution structural reference over large areas, enabling GEDI canopy cover performance to be evaluated within site variation in canopy density, vegetation structure, and landscape. Detailed information about each site, including its size, elevation, slope, and land cover, is provided in **Supplementary Table 1**.

**Figure 1:**
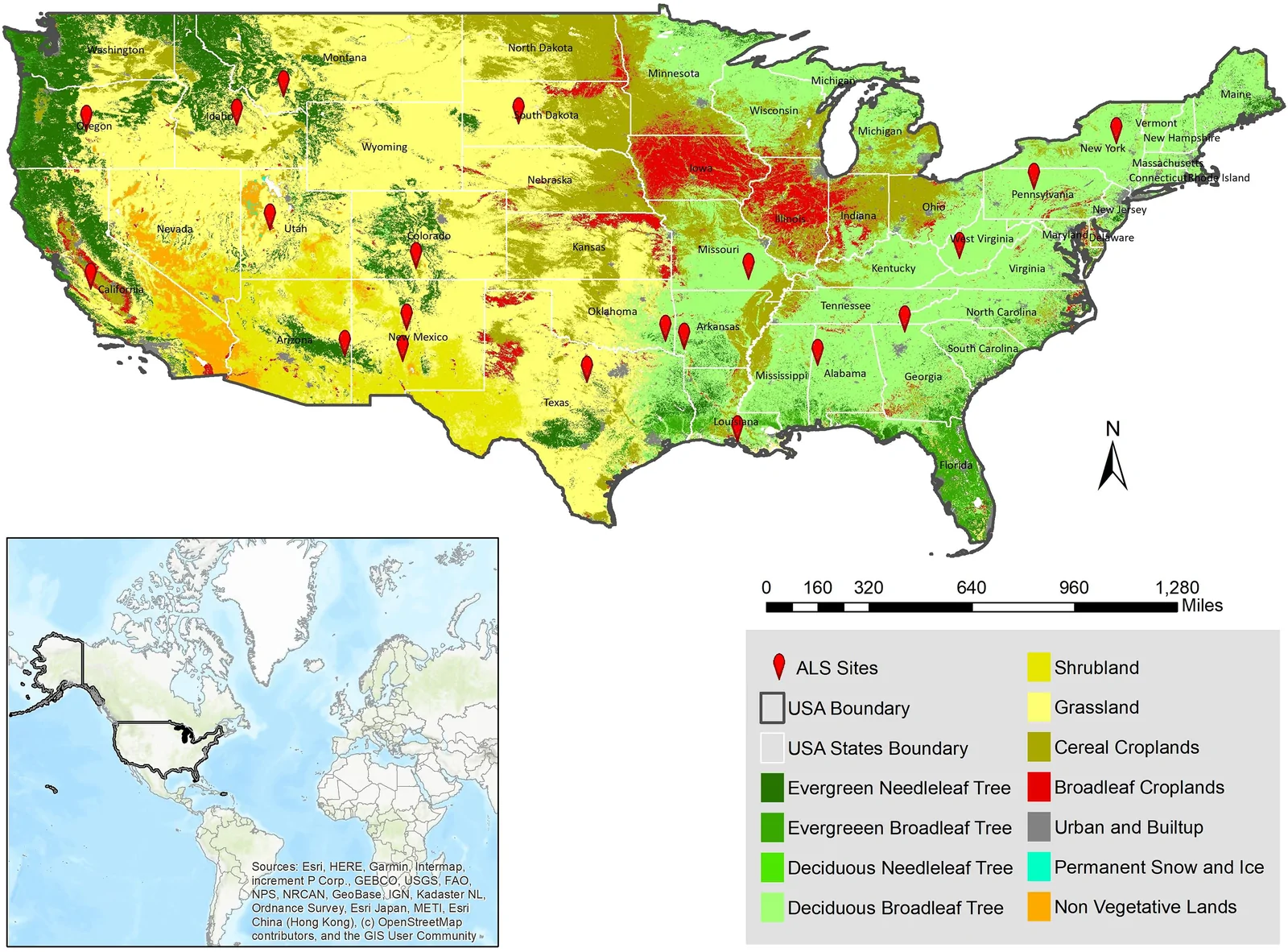
Locations of the 20 sites chosen for this investigation are indicated by the red dots. A map of land cover from MODIS (Friedl and Sulla-Menashe, 2022) serves as the background.

### 2.2 GEDI data

Version 2 of the GEDI L2B product was retrieved from the NASA data portal at Land Processes Distributed Active Archive Center (LPDAAC) (https://lpdaac.usgs.gov; accessed in April 2025). Version 2 data products have a higher degree of geolocation accuracy due to post-processing and pointing, range, and timing calibration upgrades, which are anticipated to decrease the geolocation error (1σ) from the previously reported 20 m horizontal accuracy (1σ) and 50 cm vertical accuracy to ~8 m horizontal and 10 cm vertical uncertainty (Dubayah et al., 2020).

L2B contains derived canopy metrics, including canopy cover with sensitivity metrics and quality flags for each footprint. The sensitivity flag refers to the cover density at which GEDI can reliably penetrate through to detect the surface (Dubayah et al., 2020; Tang et al., 2019), providing an index of the proportion of the waveform returned from the ground surface. The quality flags also facilitate assessment of waveform and algorithm reliability as described in the next section and in more detail in Hofton et al. (2019) and Tang et al. (2019).

#### 2.2.1 GEDI data processing

The GEDI data was filtered to eliminate waveforms that were marked as inappropriate for evaluating canopy cover (“Quality flag” = 1), flagged as detector-saturated (“Degrade flag” = 1), or had sensitivity levels below 0.98 to reduce occurrence of false positive ground returns. These filters eliminate the majority of GEDI coverage beams, observations over water bodies, and cloud-contaminated observations (Dubayah et al., 2020; Potapov et al., 2021). From a total of 800,947 GEDI footprints located within the 20 field sites, high-quality geolocated canopy cover metrics were retrieved for ~58% (464,549 footprints), implying that 42% (336,398) of measurements were identified as sub-optimal by the instrument and algorithm quality flags. The filtered GEDI datasets were projected from the World Geodetic System 1984 (WGS84) coordinates to the Universal Transverse Mercator (UTM) projection zone that corresponded to ALS coverage (WGS84: UTM = 10–19 N, EPSG = 32610–32619 and NAD83: UTM = 2–9 N, EPSG = 26910 – 26919) of the validation sites as described below.

### 2.3 Airborne Laser Scanner data

ALS derived canopy cover was used as a high resolution structural reference for comparison with GEDI L2B canopy cover estimation. ALS point clouds collected by the USGS 3D Elevation Program (3DEP) between 2017 to 2022 were acquired using the 3DEP data portal (https://apps.nationalmap.gov/downloader). The goal of the 3DEP LiDAR project is to obtain complete national airborne LiDAR coverage, with quality specifications determined by the National Enhanced Elevation Assessment (NEEA), at maximum 8-year intervals (Heidemann, 2012). The 3DEP data has already been geo-rectified, quality checked, and categorized as ground, noise, water, and vegetation. Horizontal coordinates are referenced to the Universal Transverse Mercator projection. ALS 3DEP data met or exceeded USGS quality level 2 criteria to accurately measure vegetation structure (<19.6 cm vertical accuracy, with <2 m horizontal geolocation error).

We used R software sf and lidR packages for processing ALS 3DEP data (Pebesma, 2018; R Core Team, 2010; Roussel et al., 2020). Lidar returns that were marked as overlapping two or more cloud points were removed. The lidR package points2raster technique was then used to transform height-filtered and normalized point clouds into raster-based canopy height models (CHM).

The 3DEP canopy height model (CHM) represents the height of the canopy from the ground to the topmost canopy structure at a horizontal grid spacing of 1 m. We followed three steps to create the CHM. 1) The 3DEP ALS point cloud data was first normalized using a triangular irregular network (TIN) to generate a digital terrain model (DTM) of the ground surface (Roberts and Hanan, 2024). 2) The ALS point cloud was rasterized to a 1 m grid to generate a digital surface model (DSM), with each pixel representing the highest valid elevation return within that grid cell. Before rasterization, outliers >80 m associated with low-level clouds, smoke, birds, and elevation discrepancies near water ground interfaces were removed. The DTM and DSM were generated on the same 1 m grid and with the same spatial alignment. Finally, the CHM was calculated as CHM = DSM − DTM, producing canopy height above ground at 1 m horizontal resolution and vertical precision <0.19 m.

The CHM was classified into two classes based on height (ground, grass, and herbs: <1 m and woody vegetation: >1 m height) to calculate the canopy cover (Coverdale et al., 2024; Schlickmann et al., 2025). ALS canopy cover was calculated as the proportion of pixels exceeding the selected woody vegetation height threshold within the corresponding GEDI footprint. We used a 1 m height criterion (rather than the often-used 2 m criterion) to avoid underestimation of shrubs that are frequently < 2 m tall. Shrub vegetation height in drylands is often <2 m, a higher threshold (2 m) may result in underestimation of canopy cover by excluding 1–2 m woody vegetation layer in these ecosystems (Coverdale et al., 2024; Schlickmann et al., 2025). A buffer of 12.5 m radius was computed around the center location of each GEDI footprint (and repeated at 1 meter intervals around the center point for the geolocation analysis described below). Within each GEDI footprint, canopy cover was calculated using the ALS (3DEP) CHM and compared to GEDI cover estimates. The raster data was processed using the terra, extract, and raster libraries in R (Pebesma, 2018; R Core Team, 2010; Roussel et al., 2020).

### 2.4 GEDI co-location analysis

Approaches for optimal co-location of two distinct lidar datasets have been developed in earlier research (e.g., Blair and Hofton, 1999). This so-called “bullseye” approach recognizes the uncertainty in the actual GEDI footprint location within a larger ALS coverage, which is mitigated by sampling at different angles and distances from the nominal footprint center to search for the best corresponding values in the ALS data (Li X. et al., 2024; Hancock et al., 2019).

In this study, we implemented a ‘bullseye’ approach to optimize the geolocations of the GEDI footprints. To assess the potential influence of GEDI geolocation uncertainty, we conducted a local co-location sensitivity analysis by systematically moving each GEDI footprint in relation to the ALS reference throughout a range of predefined distances and directions (Quiros et al., 2021; Tang et al., 2023; Schleich et al., 2023). To achieve this, we jittered the y and x positions of each GEDI footprint in 30 degree intervals at 5, 10, 15, 20, and 25 m of the nominal footprint center to find the optimal angle and distance (i.e., minimizing the difference between the ALS and GEDI estimates) relative to the original GEDI footprint locations. The best co-location was determined by the displacement that resulted in the smallest difference between GEDI and ALS derived canopy cover.

### 2.5 Ancillary data

We used ancillary data to evaluate the impact of factors such as climate, height, and land cover type on the precision of GEDI canopy cover retrievals. To compute the aridity index (AI), we used WorldClimate data (Fick and Hijmans, 2017). Terrain slope was retrieved from the SRTM data at 30m resolution (Kellndorfer et al., 2004). Data on land cover were derived from the MODIS land cover database (MCD12Q1 type 5, a Level 6.1 product), including information on forest types, needleleaf and broadleaf forests, and shrublands (Friedl and Sulla-Menashe, 2022).

### 2.6 Accuracy assessment

ALS data were used to assess the reliability of GEDI spaceborne (sb) measurements. We examined the azimuth dependence of geospatial offsets and contrasted the differences in geolocation offsets throughout forest types to investigate how GEDI footprint geolocation offsets varied with azimuth and forest types. We also evaluated the pre-registration accuracy of GEDI cover estimates with post-georegistration accuracy. We employed ordinary least squares (OLS) regression and standardized major axis (SMA) regression to analyze the overall relationships, with slope (s) and intercept (c) used to detect persistent biases. We also employed additional assessment metrics (root mean square error (RMSE), mean error (ME), mean absolute percentage error (MAPE), and mean relative error (MRE)) to provide additional insights about the error distributions in GEDI canopy cover retrievals.

## 3 Results

We assessed GEDI footprint level canopy cover at 20 sites by using a total of 464,549 GEDI shots. We examined the GEDI cover accuracy (through ME, MRE, and RMSE, relative to the ALS estimates) for all data combined, for arid and humid climate zones, and for broadleaf, needleleaf, and shrub plant functional types (PFT; **Table 1**; **Figures 2**–**4**).

**Table 1:** Goodness-of-fit statistics between GEDI canopy cover estimates and ALS reference data sorted by climate and forest types, with original footprint location errors (“pre-registration”) and co-colocation errors (“post-registration”) showing the impact of optimizing localization of the GEDI footprint relative to the ALS reference data in a radius of up to 12.5 m.

Mean cover (fraction)	Number of samples (N)	Pre-registration					Model	Post-registration				
		R^2^	RMSE (fraction)	MRE (%)	s	c		s	c	R^2^	RMSE (fraction)	MRE (%)
All sites												
0.69	464549	0.84	0.14	40.1	0.88	0.12	OLS	0.98	0.03	0.96	0.07	5.4
					0.97	0.05	SMA	0.99	0.01			
Humid												
0.77	267408	0.81	0.13	20.7	0.90	0.10	OLS	0.97	0.04	0.94	0.07	2.8
					0.96	0.04	SMA	0.99	0.02			
Arid												
0.51	197141	0.79	0.16	69.8	0.89	0.09	OLS	0.96	0.03	0.93	0.08	9.4
					1.01	0.06	SMA	0.98	0.001			
Broad leaf												
0.71	175518	0.82	0.14	24.9	0.91	0.10	OLS	1.02	0.01	0.94	0.06	3.4
					1.04	0.04	SMA	1.01	-0.01			
Needle leaf												
0.74	169016	0.81	0.12	25.7	0.84	0.11	OLS	0.97	0.02	0.96	0.05	2.4
					0.93	0.05	SMA	0.99	0.01			
Shrub												
0.48	120015	0.82	0.17	84.1	0.93	0.09	OLS	0.98	0.01	0.95	0.09	12.3
		1.02			0.05	SMA	0.99	0.001				

“N” provides the number of samples in each category, with other columns showing the root mean square error (RMSE) and mean relative error (MRE = (100* GEDI - ALS)/ALS), while σ and c provide the ordinary least squares (OLS) and Model-II (SMA) regression slope and intercept of the relationships GEDI ~ c and σ* ALS.

**Figure 2:**
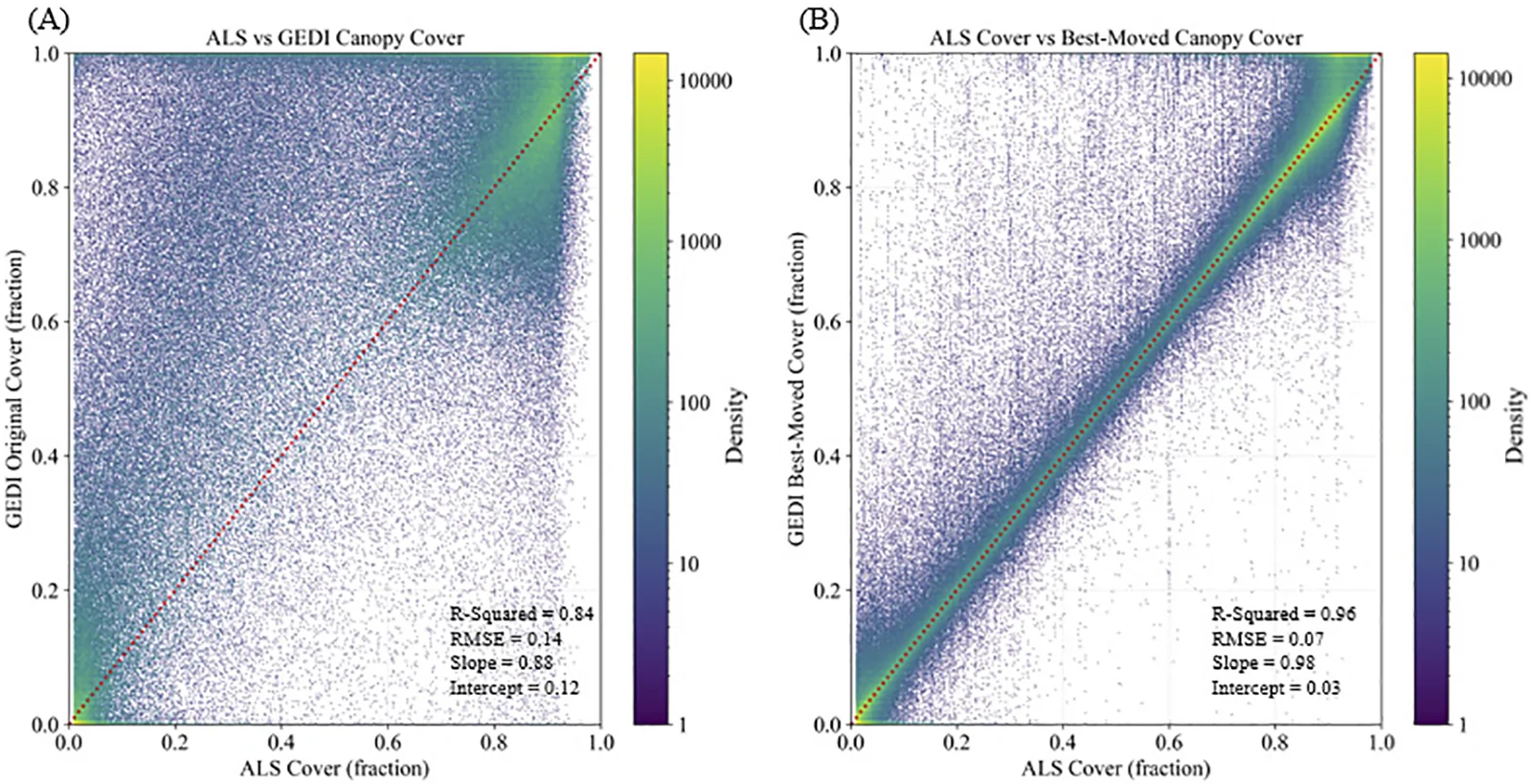
Comparison between GEDI canopy cover and USGS ALS canopy cover for all sites. **(A)** canopy cover comparison using pre-registration data, and **(B)** canopy cover comparison using post-registration data. The red dotted line represents the 1:1 line and the color scale shows the local density of sample comparisons out of a total of 464,549 GEDI footprints (see **Table 1**).

**Figure 3:**
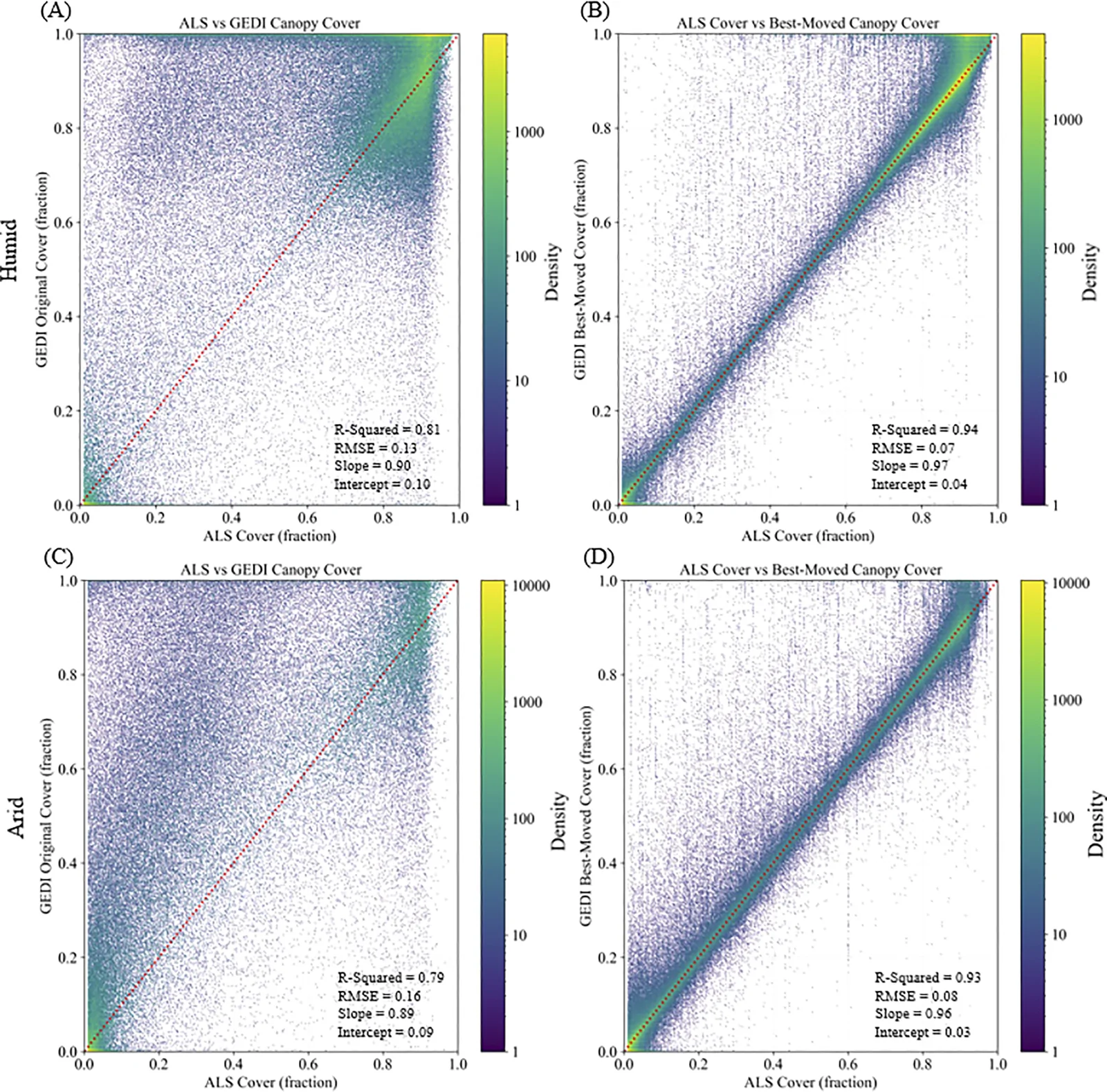
Comparison between GEDI canopy cover and USGS ALS canopy cover for arid and humid climate conditions. **(A, C)** canopy cover comparison using pre-registration data in arid and humid climates, respectively. **(B, D)** canopy cover comparison using post-registration data in arid and humid climates, respectively. The red dotted line represents the 1:1 line and the color scale shows the local density of sample comparisons.

**Figure 4:**
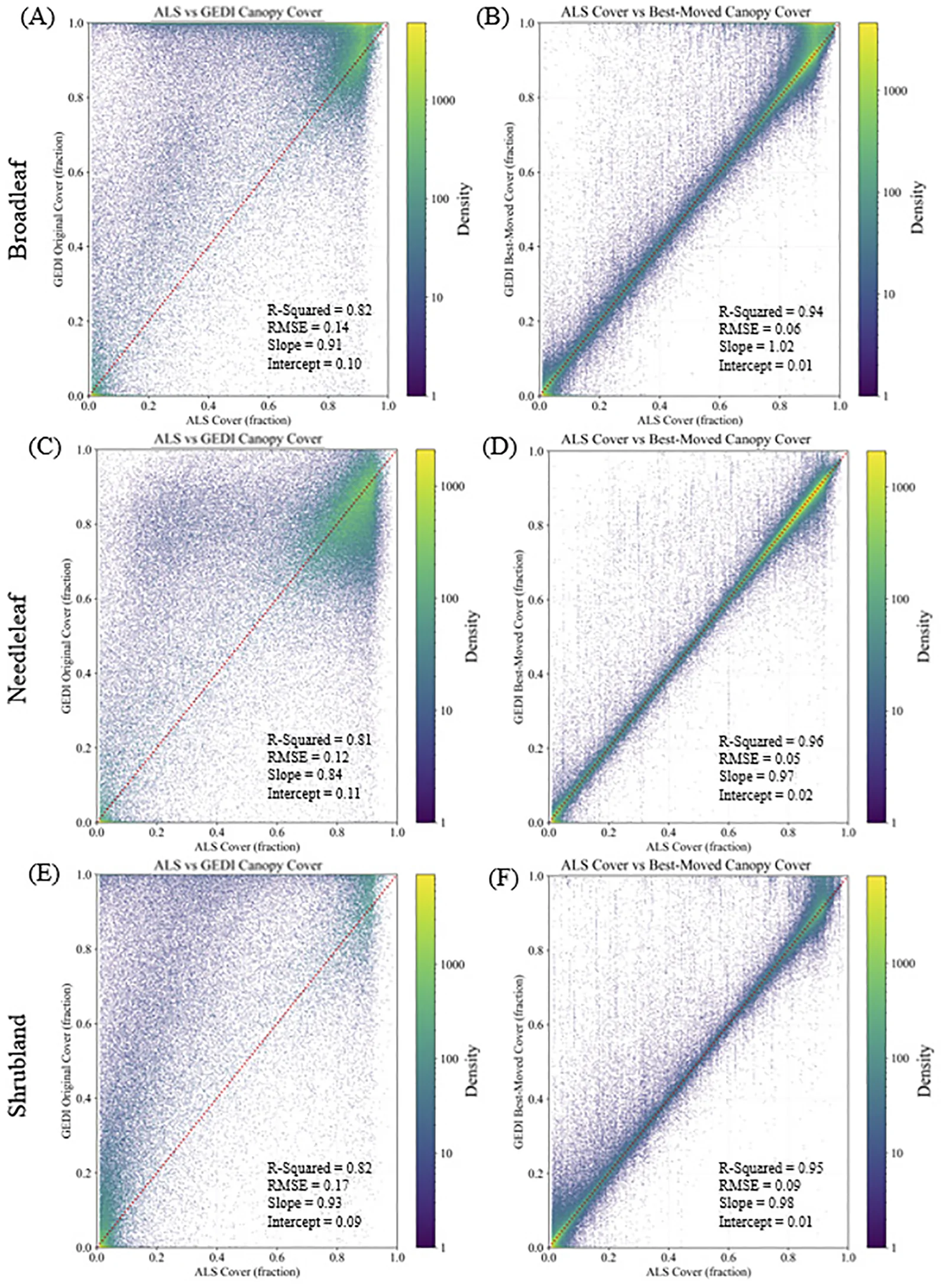
Comparison between GEDI canopy cover and USGS ALS canopy cover for different plant functional types [PFT, **(A, B)** = broadleaf, **(C, D)** = needleleaf, **(E, F)** = shrublands]. Left-side panels **(A, C, E)** show canopy cover comparison using pre-registration data and right-side panels **(B, D, F)** show canopy cover comparison for post-registration data. The red dotted line represents the 1:1 line and the color scale shows the local density of sample comparisons.

### 3.1 GEDI pre-registration vs ALS

The pre-registration GEDI ALS correlations varied considerably across 20 study sites. Across sites, R² ranged from 0.07 to 0.57 while RMSE showed a comparatively narrower range of 0.22 to 0.44 (**Supplementary Table S2**). When all GEDI footprints are pooled together, model fits indicate R^2^ = 0.84 with an RMSE = 14% (**Figure 2**, **Table 1**). GEDI estimates of canopy cover, before bullseye registration, showed good correspondence at low ALS cover (<10%) and high ALS cover (>70%), but GEDI canopy cover overestimates ALS values at intermediate covers (**Figure 2**, **Table 1**). In humid environments, which typically have more trees and higher canopy cover, the bulk of cover estimates are between 75-90% cover for both GEDI and ALS, thus overall agreement is improved (**Figure 3A**). However, even in these environments, at intermediate cover (approximately 10-70% ALS cover) GEDI cover tends to overestimate the ALS estimates. By contrast, in arid environments (**Figure 3C**), GEDI performs well at low cover (<10%) but consistently overestimates ALS cover above 10%. In the different PFT groupings, the overestimation bias at intermediate cover is particularly clear in shrublands but persists in coniferous and deciduous forests when actual cover is less than ~75% (**Figures 4A, C, E**).

### 3.2 GEDI post-registration vs ALS

Following bullseye optimum co-location (post-registration) analysis, site-level correlation increased from pre-registration at all sites, ranging from 0.17 to 0.82, while RMSE decreased to 0.12–0.33 (**Supplementary Table S2**). In the pooled footprint-level analysis, GEDI estimates of cover are also significantly improved (**Table 1**; **Figures 2**–**4** (right-hand column) and **Supplementary Figure S1**). We found ~0.14 increase in R^2^ and a 0.07 reduction in RMSE (**Figure 2B**, **Table 1**). Geolocation adjustment across all sites also resulted in a decrease of MRE from 40.1% to 0.07% (**Table 1**). Furthermore, slope and intercept also improved with a decrease in intercept and an increase in slope for both OLS and SMA regression II types (**Table 1**, **Figure 2B**). We found that GEDI height retrievals performed better across all values of cover, with the highest agreement with ALS cover occurring in high canopy cover sites (canopy cover >70%; **Figure 2B**). The difference between GEDI and ALS cover is significantly reduced by the co-location of footprints (**Figure 5**). The pre-registration overestimation of cover is reduced with the co-location of footprints, providing estimates close to the 1:1 line (**Figures 2**–**4**, right side).

**Figure 5:**
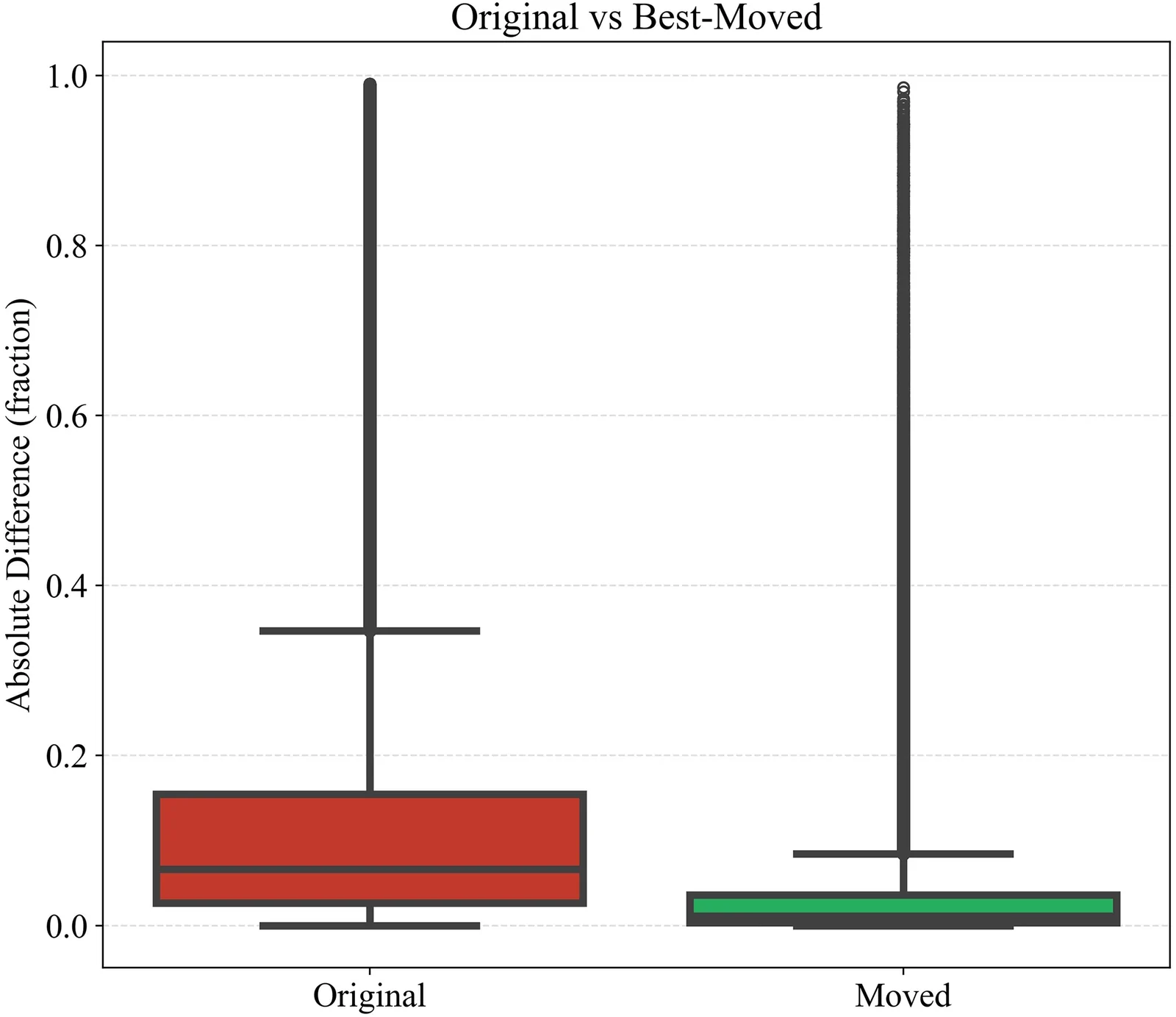
Absolute difference between original GEDI L2B canopy cover and corresponding ALS measurements, compared to the spatially optimized ALS cover. The horizontal line within each box represents the median value, while the box limits correspond to the first quartile (25th percentile) and third quartile (75th percentile). The horizontal line outside of each box represents the minimum and maximum values. Individual open circles outlined beyond the whiskers indicate outliers. The “Moved” group has a smaller median difference and more tightly clustered data than the “Original” group, but both distributions are highly right-skewed, with outliers near 1.0.

The geolocation offset analysis suggests that the optimal locations that minimize the canopy cover difference between GEDI and ALS tends to be within 5 m of the nominal footprint center, with dominant angles being toward the south and north (**Figure 6**). Distances beyond 5 m from the nominal GEDI footprint center were less frequently selected, suggesting that GEDI navigation is generally accurate to within 5 meters, but that the source of geolocation offsets tends to oscillate to the south and north of the nominal center (**Figure 6**).

**Figure 6:**
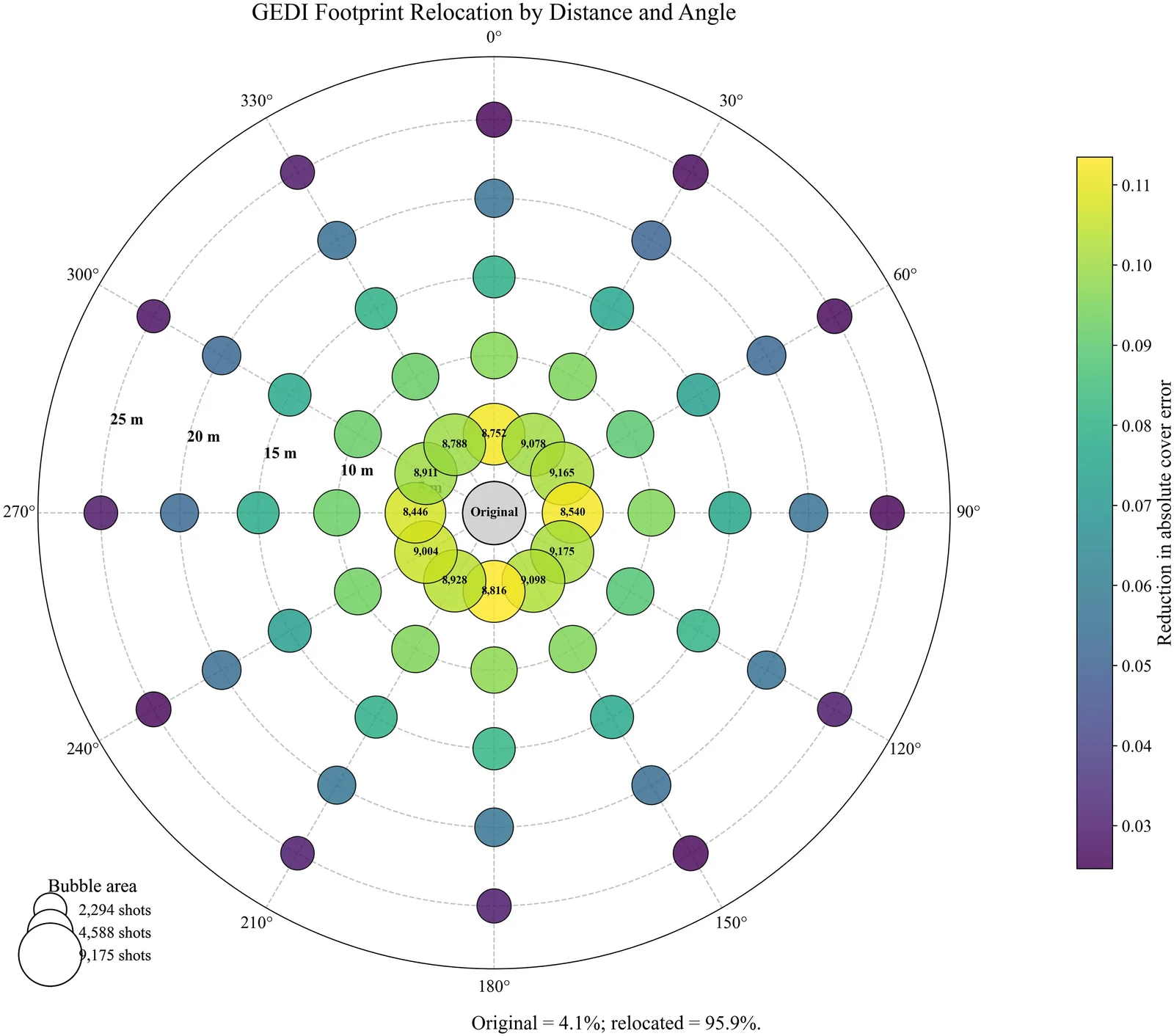
Polar rose plot of geolocation offsets for the post-registration angle of GEDI footprints for all forest sites.

### 3.3 GEDI canopy cover performance with aridity

We classified the field sites as humid or arid based on an aridity index threshold of 0.65 (Huang et al., 2016; Middleton and Thomas, 1994). As in the overall dataset, we see better performance in both arid and humid environments at low and high cover, with distinct overestimation at intermediate cover and improvements following geolocation correction (**Figure 3**, **Table 1**). In the more humid eastern United States and western coastal areas we examined 267,408 L2B footprints in 9 ALS sites with an average ASL canopy cover of 77% (**Table 1**; **Supplementary Tables S1**, **S2**). For humid region regression models, the slope increases (OLS: 0.9 to 0.97 and SMA: 0.96 to 0.99), and the intercept is reduced (OLS: 0.10 to 0.04 and SMA: 0.04 to 0.02) after the post-registration process (**Table 1**). By contrast, most of the Western US, excluding coastal areas, has a dry and hot climate (AI <0.65). These environments are distinguished by low density and low-stature (LDLS) woody vegetation. We examined 197,147 GEDI cover footprints in 11 ALS sites in arid regions (**Supplementary Table S1**), with mean canopy cover of 51% (**Table 1**) and improved cover estimates at intermediate cover following the geolocation correction (**Table 1**; **Supplementary Table S2**).

### 3.4 GEDI canopy cover performance with PFT

We assessed canopy cover at 9 broadleaf sites by using 175,518 footprints of L2B, 6 needleleaf sites by using 169,016 footprints, and 5 shrubland sites by using 120,015 footprints, as defined in the land cover dataset (**Table 1**, **Supplementary Table S2**). GEDI canopy cover estimation performance at the site level varied over PFTs (**Supplementary Table S2**). Before registration, shrub sites exhibited higher RMSE (0.33-0.44) than needleleaf (0.24-0.32) and broadleaf (0.22-0.43) sites. We found a mean canopy cover of 0.71 in broadleaf, 0.74 in needleleaf, and 0.48 in shrublands with all GEDI footprints pooled together (**Table 1**). Optimal co-location enhanced the apparent precision of GEDI cover estimates for all PFT types, resulting in a drop in RMSE of ~0.08 and an increase in R^2^ of ~0.15. After registration, all PFT types had an R^2^ greater than 0.94 and an RMSE of 0.07 ± 0.02. Slope and intercept also improved, indicating reduced bias, following geolocation for all PFT.

We found that canopy cover overestimation in broadleaf is reduced from 24.9% MRE to 3.4% (**Table 1**). In needleleaf and shrublands, overestimation reduced from 25.7% to 2.4% MRE and 84.1% to 12.3% MRE, respectively. Although it appears that the GEDI cover biases have mostly reduced, some significant biases are still present. This might be due to ambiguity in retrieval algorithms, particularly in complex forest stands. The discrepancy in time of acquisition between ALS and GEDI data may exceed two years in some cases, leading to extra uncertainty for validation. In terms of distance and angle for optimal geolocation, error improvement for all PFTs is similar, with optimal distances ~5 m and little or no trend for angle selection.

## 4 Discussion

GEDI retrieves vegetation structural factors within each footprint by capturing the radiance attenuation profile. Previous research has evaluated GEDI’s accuracy in estimating terrain height, canopy height, and leaf area index (Adam et al., 2020; Liu et al., 2021; Quiros et al., 2021; Tang et al., 2023). However, limited work has examined GEDI cover product accuracy across different eco-climate zones and by considering geolocation offset. Our evaluation of GEDI cover addressed this gap, increasing our understanding of GEDI canopy cover estimates in different eco-climate zones. Correction of the geolocation offset revealed a significant increase in agreement between GEDI and ALS cover and a decrease in error for all eco-climatic conditions. We found that, on average, a 5 m co-location correction provides better cover agreement, with shift directions concentrated at 0° (North) and 180° (South).

GEDI canopy cover estimation is largely influenced by forest structure, canopy density, geographic location, and footprint co-registration (Fayad et al., 2022; Li L. et al., 2023; Li X. et al., 2024; Li Y. et al., 2024; Mandl et al., 2023; Oliveira et al., 2023). Li Y. et al. (2024) found that GEDI canopy cover retrieval accuracy changed with canopy density and reported a canopy cover bias of around -0.11 compared to ALS. Li X. et al. (2024) observed good agreement with co-registered aerial LiDAR (RMSE = 0.10; R² = 0.85) and demonstrated effects of geolocation on cover estimation. Li X. et al. (2024) also found that GEDI geolocation offsets showed no consistent dependence on azimuth direction, suggesting that positional errors were not associated with a dominant directional bias. Our findings demonstrate a trend toward overestimation at intermediate canopy cover, suggesting that the extent and direction of GEDI canopy cover bias can vary with canopy structure, reference cover definition, topography, and footprint co-registration.

Inaccuracies in the L2B pre-registration cover product can be attributed to the GEDI waveforms cover estimating technique. Prior studies stated that the GEDI cover is underestimated, particularly for low height and open forests (Fayad et al., 2022; Mandl et al., 2023; Oliveira et al., 2023) where this study suggests that GEDI overestimates cover for intermediate cover sites. The GEDI cover algorithm is dependent on the canopy gap (Hofton et al., 2019). In canopy gap detection, the gap is primarily determined by retrievals of the canopy top height, canopy bottom and ground returns (Hofton et al., 2019). Small differences between canopy top and bottom lead to an underestimation of canopy cover in lower-height canopy areas, whereas in tall canopies height this resulted in an overestimation. GEDI ALS temporal differences may reflect actual vegetation change or disturbance rather than estimation error. For instance, differences in data acquisition dates between GEDI and ALS add another layer of uncertainty since canopy conditions may change between observation dates. Another factor contributing to significant inaccuracy in arid woods is laser ground detection capability. The poor ground returns in arid locations due to sparse vegetation reduces GEDI’s ability to capture canopy gaps and canopy cover in short vegetation. The 15.6 ns GEDI laser pulse limits the reliable separation of plant and ground returns at heights < 2.3 m (Nazir et al., 2026). Vegetation returns may overlap with the ground signal, potentially affecting GEDI waveform derived canopy cover estimations. This limitation, together with irregular canopy distribution, could factor into the lower ALS-GEDI agreement and higher uncertainty observed at some shrub dominated (height below 2.3 m) sites. Canopy cover is underestimated in high canopy cover regions and overestimated in low canopy cover regions.

In case of cover overestimation error, the main source of canopy cover overestimation might be the residual present within-crown gaps that are smaller than LiDAR footprints (Li X. et al., 2024). The decomposition technique and waveform processing are mostly responsible for the canopy cover difference. By using the GEDI beam sensitivity flag from the simulated waveform, it is possible to investigate the product uncertainties caused by noise in the GEDI observed waveform (Li X. et al., 2024). The small-footprint size of the ALS data (< 1 m) is likely to underestimate canopy cover due to inclusion of gaps within tree canopies. Despite maintaining a consistent phenological time, it should be mentioned that the ALS data were collected over several years (2017 to 2022), such that waveform matching for ALS-GEDI registration may be affected by temporal mismatch, which could increase uncertainty in cover product validation. Additionally, part of the observed difference may arise from differences in sensor sampling and retrieval methodology, as GEDI and ALS derived canopy cover estimation represent related, but not algorithmically identical, measurements. GEDI L2B canopy cover is derived from waveform energy, whereas ALS canopy cover was calculated as the proportion of CHM pixels exceeding the selected height threshold, which may contribute to systematic differences between the two estimates. For instance, a different height threshold than 1 m for ALS canopy cover could result in different canopy cover estimates within the GEDI footprint.

GEDI footprint co-location reduced canopy cover error with offsets generally <5 m and cover error increasing with spatial offsets >15 m (Li X. et al., 2024). For example, during limited time periods (October 2019 and June 2020) optimal geolocations shifts ranged 10-25 m horizontally (Tang et al., 2023). However, offsets may differ considerably between strong and coverage beams and, for the L2A height product, along-track offsets varied by about 10 m, whereas cross-track offsets varied by 5.5 m (Tang et al., 2023). In this study, we used quality flags to filter GEDI poor-quality shots and computed cover at different distances (5 to 25 m) and angles (0° to 330°). We found optimal correction distances were generally <10 m, with RMS errors increasing at larger distances.

After ALS-GEDI registration, GEDI cover and ALS cover estimates have an overall better agreement. However, GEDI cover overestimates in medium dense stands and can overestimate in tall dense stands. Additionally, earlier studies discovered that the GEDI cover was consistently low for shrublands and in open forests (<0.4 cover) (Mandl et al., 2023). The underestimation of heights in low cover ecosystems is primarily due to the challenge of decomposing ground returns from waveforms in low-height vegetation (Fayad et al., 2022; Mandl et al., 2023; Oliveira et al., 2023). By contrast, our results indicate consistent overestimation of actual canopy cover rather than underestimation, particularly in ecosystems with actual canopy cover in the range 10-70%. In this study, we used the most updated L2B product acquired in mid-2025, and we used a higher number of GEDI shots than previous studies. Our findings suggest that GEDI cover is more reliable in low and high cover situations, with a tendency to overestimate at intermediate cover, particularly for non-geolocation corrected data. Our validation findings further demonstrated the necessity of ALS geolocation throughout the validation process, particularly in forest zones with substantial heterogeneity. This finding is consistent with earlier research on ALS registration (Wu et al., 2020, 2021; Zhao et al., 2009). The findings of this study also highlight GEDI’s significant advantage in mapping canopy cover across large geographic contexts, suggesting that GEDI cover can track small variations in cover that are frequently linked to minor natural disturbances, advancements, and declines in forests.

## 5 Conclusion

The study evaluates GEDI canopy cover with high-resolution airborne laser scanning (ALS) data acquired from 20 study sites across the US. Product comparison indicates that location corrections, using the bullseye approach (Quiros et al., 2021; Tang et al., 2023; Schleich et al., 2023), improves overall results but the degree to which such corrections represent artificial improvements cannot be evaluated. We observed a correlation of 0.84 with an RMSE of 14.1% for pre-registration, while with geolocation correction R^2^ = 0.96 with an RMSE of 7.2%. In pre-registration, the primary cause of canopy cover overestimation by GEDI is likely due to inaccurate ground determination produced by waveforms and pulse width. This over estimation of cover may also be caused by measurement scale differences between GEDI and ALS cover, with smaller ALS data able to quantify smaller (~1 m) gaps within larger canopies, which must be assessed in future studies. In co-location, the GEDI cover product effectively correlates with the ALS cover product. Among the PFTs, total cover in broadleaf forests was most accurately estimated, compared to relatively sparse vegetation (shrublands) which had a lower estimation accuracy. In eco-climate zones, GEDI can offer reliable canopy cover estimates in mesic forests, but provides lower accuracy in drylands. This investigation provides baseline error assessments for GEDI canopy cover estimates across the diverse plant functional types and climate zones of CONUS that can help with product development and enhance the general understanding of the GEDI cover product.

## Data Availability

Publicly available datasets were analyzed in this study. This data can be found here: https://search.earthdata.nasa.gov/search.
