# Supplementary material for: GEDI canopy cover assessment using airborne LiDAR across eco-climate regions of the United States
Source: Front Plant Sci. 2026 Sep 29;17:1951245. doi: 10.3389/fpls.2026.1951245 (PMC13658151; doi:10.3389/fpls.2026.1951245)
Supplement: Supplementary file 1 [file Table1.docx]

**Supplementary Material**

**Tables**

Table S1. Details on 20 study sites across the Continental USA used in this research. Climate types are defined by the aridity index (AI, reference) with Arid climates defined by AI < 0.65 and humid climates AI > 0.65. Plant functional types (PFT) include broadleaf forest (BLF), needleleaf forests (NLF), and shrublands (Shrub). Study sites vary in area based on local vegetation similarity, avoiding agricultural and urban development.

| **Site** | **State** | **Climate Type** | **PFT Type** | **Area (km^2^)** | **Longitude (W)** | **Latitude (N)** | **Mean Annual Precipitation (mm/y)** | **Mean Annual Temperature (**°C**)** | **Altitude (m ASL)** | **Aridity Index (AI)** |
| --- | --- | --- | --- | --- | --- | --- | --- | --- | --- | --- |
| 1 | Alabama | Humid | BLF | 784 | -88.031 | 33.715 | 1483 | 16.1 | 95 | 4.27 |
| 2 | Arkansas | Humid | BLF | 610 | -94.051 | 34.435 | 1461 | 14.9 | 439 | 4.40 |
| 3 | Louisiana | Humid | BLF | 558 | -91.639 | 30.261 | 1506 | 19.5 | 10 | 4.19 |
| 4 | Missouri | Humid | BLF | 2322 | -91.141 | 37.583 | 1090 | 12.9 | 414 | 3.67 |
| 5 | Tennessee | Humid | NLF | 446 | -84.102 | 35.227 | 1576 | 12.2 | 685 | 4.86 |
| 6 | New York | Humid | BLF | 761 | -74.544 | 43.702 | 1296 | 3.6 | 797 | 7.12 |
| 7 | Oklahoma | Humid | BLF | 561 | -94.888 | 34.804 | 1248 | 15.9 | 260 | 3.70 |
| 8 | Pennsylvania | Humid | NLF | 314 | -78.271 | 41.649 | 1074 | 7 | 584 | 4.51 |
| 9 | West Virginia | Humid | NLF | 958 | -81.632 | 38.526 | 1100 | 12.4 | 193 | 5.05 |
| 10 | Arizona | Arid | BLF | 380 | -109.356 | 34.113 | 296 | 8.4 | 2206 | 0.65 |
| 11 | California | Arid | Shrub | 470 | -120.815 | 37.150 | 240 | 16.6 | 29 | 0.58 |
| 12 | Colorado | Arid | Shrub | 440 | -106.145 | 38.074 | 232 | 5.9 | 2344 | 0.64 |
| 13 | Idaho | Arid | BLF | 512 | -114.222 | 44.538 | 230 | 6.4 | 1533 | 0.60 |
| 14 | Montana | Arid | NLF | 430 | -112.116 | 45.821 | 249 | 6.5 | 1330 | 0.65 |
| 15 | New Mexico 1 | Arid | Shrub | 614 | -106.743 | 33.892 | 111 | 13.1 | 1522 | 0.20 |
| 16 | New Mexico 2 | Arid | Shrub | 524 | -106.561 | 35.297 | 131 | 13.2 | 1535 | 0.28 |
| 17 | Oregon | Arid | NLF | 338 | -121.021 | 44.256 | 228 | 8.3 | 935 | 0.55 |
| 18 | South Dakota | Arid | NLF | 350 | -101.518 | 44.607 | 314 | 8.3 | 536 | 0.64 |
| 19 | Texas | Arid | Shrub | 990 | -98.4310 | 32.941 | 250 | 17.9 | 304 | 0.64 |
| 20 | Utah | Arid | BLF | 420 | -112.736 | 39.809 | 201 | 10.3 | 1413 | 0.62 |

Table S2. Details on canopy cover validation (pre-registration) and co-location (bullseye) analysis across 20 study sites.

| **Site** | **State** | **PFT Type** | **Mean Cover (fraction)** | **Pre-registration (R^2^)** | **Pre-registration (RMSE)** | **Co-location (R^2^)** | **Co-location (RMSE)** |
| --- | --- | --- | --- | --- | --- | --- | --- |
| 1 | Alabama | BLF | 0.61 | 0.57 | 0.24 | 0.80 | 0.15 |
| 2 | Arkansas | BLF | 0.71 | 0.26 | 0.24 | 0.59 | 0.17 |
| 3 | Louisiana | BLF | 0.62 | 0.07 | 0.43 | 0.43 | 0.25 |
| 4 | Missouri | BLF | 0.65 | 0.52 | 0.22 | 0.82 | 0.14 |
| 5 | Tennessee | NLF | 0.69 | 0.34 | 0.24 | 0.81 | 0.12 |
| 6 | New York | BLF | 0.71 | 0.11 | 0.36 | 0.17 | 0.33 |
| 7 | Oklahoma | BLF | 0.61 | 0.32 | 0.30 | 0.54 | 0.22 |
| 8 | Pennsylvania | NLF | 0.69 | 0.36 | 0.26 | 0.62 | 0.19 |
| 9 | West Virginia | NLF | 0.65 | 0.31 | 0.25 | 0.77 | 0.14 |
| 10 | Arizona | BLF | 0.32 | 0.28 | 0.38 | 0.77 | 0.13 |
| 11 | California | Shrub | 0.46 | 0.15 | 0.44 | 0.39 | 0.32 |
| 12 | Colorado | Shrub | 0.36 | 0.39 | 0.33 | 0.72 | 0.17 |
| 13 | Idaho | BLF | 0.28 | 0.25 | 0.35 | 0.72 | 0.14 |
| 14 | Montana | NLF | 0.54 | 0.19 | 0.32 | 0.66 | 0.18 |
| 15 | New Mexico 1 | Shrub | 0.14 | 0.39 | 0.43 | 0.51 | 0.28 |
| 16 | New Mexico 2 | Shrub | 0.12 | 0.36 | 0.38 | 0.56 | 0.21 |
| 17 | Oregon | NLF | 0.58 | 0.21 | 0.29 | 0.69 | 0.17 |
| 18 | South Dakota | NLF | 0.39 | 0.26 | 0.31 | 0.76 | 0.15 |
| 19 | Texas | Shrub | 0.16 | 0.42 | 0.39 | 0.49 | 0.23 |
| 20 | Utah | BLF | 0.33 | 0.25 | 0.31 | 0.74 | 0.16 |

**Figures**

Figure S1: Original GEDI L2B cover vs moved canopy cover scatterplot. The color scheme shows the density of points. The red line shows a 1:1 relationship.
